# Multi-Omic Age Associations Identified With Artificial Intelligence (AI)

**DOI:** 10.1093/geroni/igaf122.473

**Published:** 2025-12-31

**Authors:** Daniel Evans, Jiashun Zheng, Peggy Cawthon, Steven Cummings, Hao Li

**Affiliations:** University of California, San Francisco, San Francisco, California, United States; University of California, San Francisco, San Francisco, California, United States; California Pacific Medical Center, San Francisco, California, United States; California Pacific Medical Center, San Francisco, California, United States; University of California, San Francisco, San Francisco, California, United States

## Abstract

There is substantial interest in using Machine Learning (ML) and Artificial Intelligence (AI) approaches to integrate multi-omics data to identify molecular factors associated with age. Multi-omic AI models can be used to predict “biological age” and to identify age-associated molecular features. To-date, predictions from traditional ML methods like elastic-net have outperformed AI models like deep learning with tabular (spreadsheet-like) data. However, new developments in transformer architectures have led to AI models that can outperform traditional ML methods. In this work, a tabular foundation AI model (TabPFN) built on transformer-based in-context learning (ICL) algorithms is used to integrate proteomics and metabolomics to identify models for age in a cohort study of older men, the Osteoporotic Fractures in Men (MrOS) Study. The serum proteomics assay was the SomaLogic 7K panel, and the serum metabolomics assay was the Metabolon Discovery HD platform. In 493 male participants split into training and test partitions, TabPFN models for age adjusted for body mass index (BMI) and estimated glomerular filtration rate (eGFR) achieved a root mean square error (RMSE) in the test set of 2.75 years, which outperformed an elastic-net model RMSE in the test set of 3.1 years. Proteins and metabolites contributing to the TabPFN model for age identified by Shapley Additive exPlanations (SHAP) included Pleiotrophin, Frizzled-7, secreted frizzled-related protein 1, and N-acetylcarnosine. Multi-omic AI models outperformed traditional ML models for age and identified biologically relevant features. Future directions include replication of findings in other studies and assessment of model performance in females.

